# No association of serum ferritin levels with advanced liver fibrosis in untreated German patients with autoimmune hepatitis

**DOI:** 10.1186/s12876-022-02588-0

**Published:** 2022-12-19

**Authors:** Bastian Engel, Elmar Jaeckel, Richard Taubert

**Affiliations:** 1 Department of Gastroenterology, Hepatology and Endocrinology, Hannover Medical School, Carl-Neuberg-Street 1, 30625 Hannover, Germany; 2 Member of the European Reference Network on Hepatological Diseases (ERN RARE-LIVER), Hannover, Germany; 3 Present Address: Ajmera Transplant Center, Toronto General Hospital, United Health Network, University of Toronto, Toronto, Canada

**Keywords:** Biochemical remission, Incomplete response, AIH, Iron, Treatment response

## Abstract

In the January 2022 issue of BMC Gastroenterology, Chen et al. report an association between serum ferritin levels and the presence of advanced liver fibrosis in patients with treatment-naïve autoimmune hepatitis (AIH). The odds ratio for ferritin in this study was marginally above 1.0. We analyzed our own published data from a German cohort for an association between ferritin levels and the presence of advanced fibrosis in treatment-naïve AIH and were not able to validate the findings of Cheng et al.

**Supplementary Information:**

The online version contains supplementary material available at 10.1186/s12876-022-02588-0.

To the editor,

We read with greatest interest the recent work by Chen et al. in BMC Gastroenterology [1] that reported a correlation of parameters of iron metabolism with advanced fibrosis in patients with untreated autoimmune hepatitis (AIH). Chen and colleagues identified ferritin levels above 199 µg/L as a non-invasive predictor of advanced fibrosis (≥ F3) in baseline biopsies from pre-treatment AIH patients in a Chinese retrospective single-center study (n = 97). Although ferritin was slightly associated with advanced fibrosis according to the odds ratio (OR) (95% confidence interval (95%-CI) 1.001–1.004), the association according to the area under the curve was moderate (AUC = 0.738, 95%-CI 0.639–0.822).

We analyzed parameters of iron homeostasis in a similarly sized retrospective single-center cohort of untreated German AIH patients [2]. As we did not investigate an association between ferritin and advanced fibrosis in the initial publication, we reanalyzed our data to investigate the findings of Chen et al. in our cohort. A detailed histological scoring of fibrosis was available from 85 of 109 patients in our cohort (Table 1). We defined advanced fibrosis as F4–F6 according to Ishak et al. [3], which should correspond to fibrosis stages F3–F4 according to Batts/Ludwig [4] as applied by Chen et al. We replicated univariate binary logistic regression (“Enter”-method, IBM SPSS Statistics 27) but found no predictive capacity of ferritin for advanced fibrosis (ferritin normalized to the upper limit of normal (ULN) OR: 1.917; 95%-CI 0.943–1.096; *p* = 0.665 or ferritin as absolute values in µg/L: OR: 1.000; 95%-CI 1.000–1.000; *p* = 0.963). This was reflected in the ROC analysis with AUCs of 0.574 (95%-CI 0.441–0.708; *p* = 0.253; absolute ferritin values) and 0.556 (95%-CI 0.424–0.689; *p* = 0.386; ferritin values normalized to the ULN) respectively in our German cohort (Fig. 1).We generally prefer to use ferritin values normalized to the upper limit of normal as this approach corrects for differences in the normal range related to sex and age which Chen et al. accounted for by including these parameters in the logistic regression analysis. To allow comparison and fair validation, we present both absolute and normalized ferritin values. Of note, AIH has different genetic and environmental risk factors in different age groups and different parts of the world, which could explain differences in clinical presentation at the time point of diagnosis of AIH, i.e. in terms of severity which may affect ferritin levels, too [5–7].

**Table 1:** Clinical parameters of patients with or without significant liver fibrosis

	F0-3	F4-6	*p*-value
Patient number	53	32	
Female sex	37 (69.8)	19 (59.4)	0.353
Age [years]	52 (17–78)	59 (20–83)	0.231
Ferritin [µg/l]	423 (15–5869)	755.0 (25.0–7892.0)	0.253
Ferritin [xULN]	1.3 (0.1–16.1)	2.5 (0.1–21.6)	0.386
TIBC [µmol/l]	67 (42–378) (n = 39)	50.0 (24.0–97.0) (n = 24)	** < 0.001**
Transferrinsaturation [%]	35.5 (16.0–100.0) (n = 40)	45.5 (16.0–100.0) (n = 24)	0.282
Iron [xULN]	1.1 (0.5–3.7) (n = 42)	1.0 (0.2–2.3) (n = 26)	0.767
IgG [xULN]	1.2 (0.5–3.6)	1.7 (0.6–4.6)	** < 0.001**
CRP [mg/l]	5.0 (1.0–38.0) (n = 51)	11.5 (1.0–77.0) (n = 28)	0.066
Hb [g/dl]	13.6 (11.4–16.5)	13.1 (9.8–15.8)	0.091
ANA	44 (84.6) (n = 52)	31 (96.9)	0.143
SMA	44 (83.0)	23 (79.3) (n = 29)	0.768
SLA	2 (3.8)	2 (6.3)	0.630
LKM	1 (1.9)	0 (0)	1.0
AIH Score	13 (10–21)	14 (10–21)	0.728
ALT [xULN]	20.8 (0.7–68.5)	15.9 (1.9–118.4)	0.207
AST [xULN]	19.1 (1.2–45.1)	17.2 (2.1–113.2)	0.964
gGT [xULN]	3.6 (0.8–23.9)	5.5 (0.5–34.1)	0.113
ALP [xULN]	1.2 (0.3–5.5)	1.4 (0.5–5.7) (n = 31)	0.131
Bilirubin [xULN]	2.1 (0.3–27.8) (n = 51)	3.7 (0.3–45.2) (n = 31)	0.207
PT [%]	78 (44–112)	63 (39–100) (n = 31)	**0.002**
mHAI	9 (4–15) (n = 49)	8 (3–15) (n = 24)	0.214
Ishak F	1 (0–3)	5 (4–6)	** < 0.001**

Fisher’s Exact test was used to compare categorical variables and the Mann–Whitney-U test was used to compare continuous variables. Results are displayed as n (%) or median (range) as appropriate. *xULN* times upper limit of normal; *TIBC* total iron binding capacity; *IgG* immunoglobulin G; *CRP* C-reactive protein; *Hb* hemoglobin; *ANA* antinuclear antibodies; *anti-SMA* anti-smooth muscle actin antibodies; *anti-SLA* anti-soluble liver antigen antibodies; *anti-LKM* anti-liver kidney microsomal antibodies; *ALT* alanine aminotransferase; *AST* asparatate aminotransferase; *gGT* gamma-glutamyl transferase; *ALP* alkaline phosphatase; *PT* prothrombin time; *mHAI* modified histologic activity index; *Ishak F* Fibrosis staging according to Ishak et al

*p*-values < 0.05 were regarded as significant and are designated in bold

**Fig. 1:**
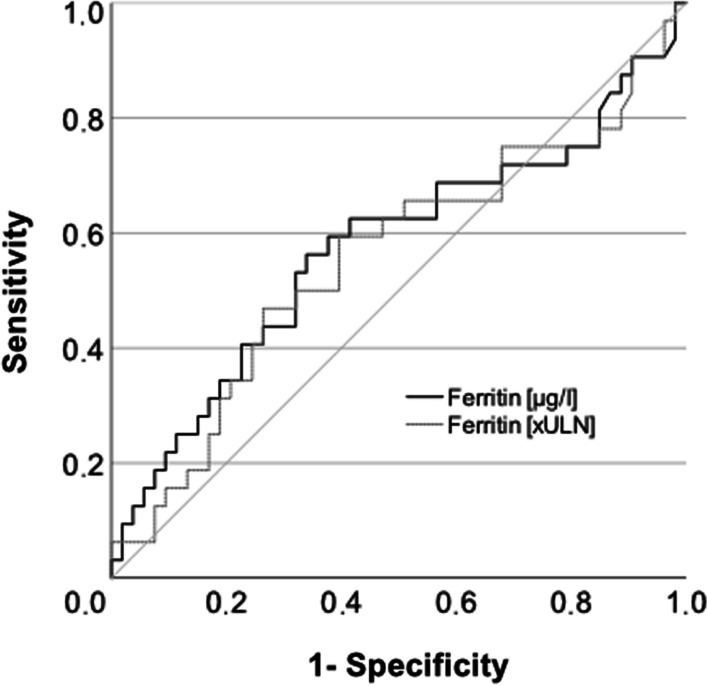
Area under the curve for the association of absolute and normalized ferritin values and advanced fibrosis. *xULN* times upper limit of normal

Next, we applied the cut-off value for ferritin of 199 µg/l from Chen et al. to divide our cohort into two groups. Remarkably, the parameters of iron metabolism were significantly different between both groups. In addition, parameters of general (C-reactive protein) and liver-specific inflammation (transaminases, cholestasis parameters) were higher in patients with higher ferritin levels. However, the histological fibrosis stages were not significantly different with regard to patients’ ferritin levels (Additional file 1: Table S1). The OR for advanced fibrosis using absolute ferritin levels with a cut-off of 199 µg/l as published by Chen et al. was 1.444 (95%-CI 0.571–3.653) (calculated using a crosstab). Overall, as the findings of Chen et al. and ours contradict each other, further analysis in other cohorts is needed to clarify the relevance of ferritin levels in terms of fibrosis prediction in AIH patients.

The main finding of our analysis of iron homeostasis in untreated AIH patients was the association of elevated ferritin levels (> 2.09 × ULN) with achieving biochemical remission after initiation of immunosuppressive therapy. In addition to elevated ferritin levels, hypergammaglobulinemia below 1.89 xULN was independently associated with achievement of biochemical remission. Finally, we could develop a treatment response score for AIH using both baseline parameters (ferritin and immunoglobulin G (IgG)). This treatment-response score was significantly associated with the achievement of biochemical remission in a training (AUC: 0.75; 95% CI 0.64–0.86) and validation cohort (AUC: 0.74; 95% CI 0.56–0.92). While almost all patients with a score < 1 achieved biochemical remission to standard of care, 20–40% of patients with a score ≥ 1 did not achieve biochemical remission. The advantage of this treatment-response score is its ability at baseline to predict achievement of biochemical remission, while a more recent treatment response score developed in a European retrospective multicenter study used the 80% decline of aspartate aminotransferase after eight weeks of therapy as predictor of biochemical remission at six and twelve months after diagnosis[8]. Interestingly, the predictive capacity of the 80% AST decline was comparable to our treatment response score (AUC (80% AST decline) = 0.65; 95%-CI 0.59–0.71).

Many centers do not measure parameters of iron homeostasis in the initial assessment of acute hepatitis, including AIH, which explains why our treatment response score has not yet been validated. Unfortunately, Chen et al. did not analyze predictors of treatment response in their Chinese cohort. Although we could not replicate the findings by Chen et al., we would be very interested to see if the prediction of treatment response in AIH by baseline ferritin and baseline IgG before starting therapy could be validated in their Chinese cohort.

## Supplementary Information

**Additional file 1**. **Table S1** Patients’ characteristics stratified by ferritin levels

## Data Availability

The datasets supporting this article are available from the corresponding author on reasonable request.

## Abbreviations

AIH: Autoimmune hepatitis
ALP: Alkaline phosphatase
ALT: Alanine aminotransferase
ANA: Antinuclear antibodies
anti-LKM: Anti-liver kidney microsomal antibodies
anti-SLA: Anti-soluble liver antigen antibodies
anti-SMA: Anti-smooth muscle actin antibodies
AST: Aspartate aminotransferase
CI: Confidence interval
CRP: C-reactive protein
gGT: Gamma-glutamyl transferase
Hb: Hemoglobin
IgG: Immunoglobulin G
Ishak F: Fibrosis staging according to Ishak et al.
mHAI: Modified histologic activity index
OR: Odds ratio
PT: Prothrombin time
TIBC: Total iron binding capacity
xULN: Times upper limit of normal
