# Supplementary material for: No association of serum ferritin levels with advanced liver fibrosis in untreated German patients with autoimmune hepatitis
Source: BMC Gastroenterol. 2022 Dec 19;22:528. doi: 10.1186/s12876-022-02588-0 (PMC9764512; doi:10.1186/s12876-022-02588-0)
Supplement: Supplementary file 1 — Additional file 1. Table S1 Patients’ characteristics stratified by ferritin levels [file 12876_2022_2588_MOESM1_ESM.docx]

**Supplemental table 1: Patients’ characteristics stratified by ferritin levels**

|  | Ferritin ≤ 199 µg/l | Ferritin > 199µg/l | p-value |
| --- | --- | --- | --- |
| Patient number | 31 | 54 |  |
| female sex | 24 (77.4) | 32 (59.3) | 0.102 |
| Age [years] | 54 (17 - 72) | 56 (20 - 83) | 0.242 |
| Ferritin [µg/l] | 104 (15 - 178) | 942.5 (203.0 - 7892) | **< 0.001** |
| Ferritin [xULN] | 0.3 (0.1 - 1.3) | 3.6 (0.6 - 21.6) | **< 0.001** |
| TIBC [µmol/l] | 70 (49 - 378) (n=20) | 57 (24 - 98) (n=43) | **< 0.001** |
| Transferrinsaturation [%] | 30 (16 - 55) (n=21) | 54 (16 - 100) (n=43) | **< 0.001** |
| Iron [xULN] | 0.8 (0.4 - 3.7) | 1.3 (0.2 - 2.3) (n=47) | **0.001** |
| IgG [xULN] | 1.3 (0.6 - 4.6) | 1.4 (0.5 - 3.6) | 0.742 |
| CRP [mg/l] | 4.0 (1.0 - 18.0) (n=27) | 11.5 (1.0 - 77.0) (n=52) | **< 0.001** |
| Hb [g/dl] | 13.5 (9.8 - 15.7) | 13.5 (11.4 - 16.5) | 0.938 |
| ANA | 28 (90.3) | 47 (88.7) (n=53) | 1.0 |
| SMA | 23 (74.2) (n=30) | 44 (84.6) (n=52) | 0.389 |
| SLA | 2 (6.5) | 2 (3.7) | 0.620 |
| LKM | 1 (3.2) | 0 (0) | 0.365 |
| AIH Score | 12 (10 - 21) | 14 (10 - 21) | 0.628 |
| ALT [xULN] | 7.5 (0.7 - 49.8) | 23.0 (1.9 - 118.4) | **< 0.001** |
| AST [xULN] | 5.0 (1.2 - 53.9) | 23.8 (2.1 - 113.2) | **< 0.001** |
| gGT [xULN] | 2.6 (0.5 - 16.5) | 5.1 (0.8 - 34.1) | **0.005** |
| ALP [xULN] | 1.0 (0.3 - 3.3) | 1.4 (0.5 - 5.7) (n=53) | **0.003** |
| Bilirubin [xULN] | 1.4 (0.3 - 25.1) (n=30) | 7.7 (0.6 - 45.2) (n=52) | **< 0.001** |
| PT [%] | 86 (47 - 100) | 68 (39 - 112) (n=53) | **0.002** |
| mHAI | 8 (4 - 15) (n=27) | 9 (3 - 15) (n=46) | 0.074 |
| Ishak F | 2 (0 - 6) | 3 (0 - 6) | 0.452 |

Fisher’s Exact test was used to compare categorical variables and the Mann-Whitney-U test was used to compare continuous variables. Results are displayed as n (%) or median (range) as appropriate. xULN: times upper limit of normal; TIBC: total iron binding capacity; IgG: immunoglobulin G; CRP: C-reactive protein; Hb: hemoglobin; ANA: antinuclear antibodies; anti-SMA: anti-smooth muscle actin antibodies; anti-SLA: anti-soluble liver antigen antibodies; anti-LKM: anti-liver kidney microsomal antibodies; ALT: alanine aminotransferase; AST: asparatate aminotransferase; gGT: gamma-glutamyl transferase; ALP: alkaline phosphatase; PT: prothrombin time; mHAI: modified histologic activity index; Ishak F: Fibrosis staging according to Ishak et al.
